# Supplementary material for: Capacity to provide care for common childhood infections at low-level private health facilities in Western, Uganda
Source: PLoS One. 2021 Oct 20;16(10):e0257851. doi: 10.1371/journal.pone.0257851 (PMC8528317; doi:10.1371/journal.pone.0257851)
Supplement: S2 File — (PDF) [file pone.0257851.s002.pdf]

Quality of care for common childhood infections study

Participant number: |____|_____|______|

Highest level of Training: Tick one that applies

Nursing assistant	Certificate in Nurse	Diploma in nurse/midwifery	Degree nurse/midwife	Diploma in Clinical medicine	Doctor (MBChB)	Master degree in paediatrics or other clinical discipline

Answer with 0 if you strongly disagree, 1 if you are neither agree nor disagree 2 if you strongly agree	
1. A 2-year-old with diarrhoea for 10 days, eating poorly and vomits feeds given to her since this morning. On examination, she is weak but alert. The following should be done in managing this child	
Insert a cannula and give IV ceftriaxone	
Start oral Ciprofloxin immediately	
Help the caregiver to administer ORS immediately	
Complete assessment for dehydration	
Refer the child to hospital immediately	

Answer with 0 if you strongly disagree, 1 if you are neither agree nor disagree 2 if you strongly agree	
2. Concerning diarrhoea with blood in the stool	
It may become persistent	
It is only caused by infections	
A child cannot become dehydrated	
Refer the child to hospital immediately	
It may require an antibiotic	

Quality of care for common childhood infections study

Participant number: |____|_____|______|

Highest level of Training: Tick one that applies

Nursing assistant	Certificate in Nurse	Diploma in nurse/midwifery	Degree nurse/midwife	Diploma in Clinical medicine	Doctor (MBChB)	Master degree in paediatrics or other clinical discipline

Answer with 0 if you strongly disagree, 1 if you are neither agree nor disagree 2 if you strongly agree	
3. A 10-day-old infant is brought to your clinic because she is not breastfeeding for 2 days now. She looks weak and pale. Her temperature is 38 ^{0}c. This infant needs	
Urgent referral to hospital or admission for more advanced care	
Syrup Amoxicillin and send her back home to return after 5 days	
Rectal paracetamol 250mg and encourage mother to breastfeed	
IM Quinine and ask mother to bring the baby back for another injection every day for 7 days	
Refer for false teeth extraction	

Answer with 0 if you strongly disagree, 1 if you are neither agree nor disagree 2 if you strongly agree	
4. A new born infant aged 5days old brought to your clinic because he passed 2 loose motions last night. He has no fever and breastfeeding well. The care for this baby should include	
Ask mother if the infant was immunised.	
Giving measles vaccination	
Encourage mother to continue breastfeeding	
Talk to mother to keep baby warm	
Stool microscopy since the infant has diarrhoea	

Quality of care for common childhood infections study

Participant number: |____|_____|______|

Highest level of Training: Tick one that applies

Nursing assistant	Certificate in Nurse	Diploma in nurse/midwifery	Degree nurse/midwife	Diploma in Clinical medicine	Doctor (MBChB)	Master degree in paediatrics or other clinical discipline

Answer with 0 if you strongly disagree, 1 if you are neither agree nor disagree<br>2 if you strongly agree	
5. A 2 week old has fast breathing if he is breathing:	
50 breaths per minute or more, counted twice	
60 breaths or more per minute, and the same in a second count	
30 breaths per minute, counted twice	
40 breaths per minute counted twice	
40 breaths per minute counted thrice	

Answer with 0 if you strongly disagree, 1 if you are neither agree nor disagree<br>2 if you strongly agree	
6. A 15-moths-old boy is brought to clinic with a cough which has lasted 3 days. On examination he is alert, temperature of 37.8 0c, Respiratory rate of 56 breaths but no chest in drawing. This child needs	
Urgent referral for oxygen	
IV benzyl penicillin injections 6 hourly for 5 days	
Referral for chest X-ray	
Paracetamol for fever and advise on feeding	
Ask about the child’s immunisation status as well	

Quality of care for common childhood infections study

Participant number: |____|_____|______|

Highest level of Training: Tick one that applies

Nursing assistant	Certificate in Nurse	Diploma in nurse/midwifery	Degree nurse/midwife	Diploma in Clinical medicine	Doctor (MBChB)	Master degree in paediatrics or other clinical discipline

Answer with 0 if you strongly disagree, 1 if you are neither agree nor disagree 2 if you strongly agree	
7. Concerning a child with a cough:	
You will only assess a child for a cough or difficult breathing if the caregiver specifically says that cough is the reason they came to the clinic.	
A child with a cough but no signs of pneumonia should be treated with Cotrimoxazole	
A child with a cough but no signs of pneumonia can be treated at home	
Oral antibiotics can be given to children with pneumonia	
It is best to count breaths for fast breathing when the child is active and moving around so that he does not cry.	

Answer with 0 if you strongly disagree, 1 if you are neither agree nor disagree 2 if you strongly agree	
8. Stella is a 4 year old with fever for 3 days. Today she collapsed and was rushed to your clinic. On examination, she cannot respond to her name or when pinched, she feels hot on touch, she is pale. Her respiratory rate is 62 breaths per minute. Concerning this child	
She has a danger sign	
The care includes giving an injection for malaria and refer to hospital immediately	
She is in high risk of dying	
Ask the mother to give paracetamol tablets immediately	
She should receive treatment for pneumonia immediately	

Quality of care for common childhood infections study

Participant number: |____|_____|______|

Highest level of Training: Tick one that applies

Nursing assistant	Certificate in Nurse	Diploma in nurse/midwifery	Degree nurse/midwife	Diploma in Clinical medicine	Doctor (MBChB)	Master degree in paediatrics or other clinical discipline

Answer with 0 if you strongly disagree, 1 if you are neither agree nor disagree 2 if you strongly agree	
9. In high risk malaria areas like Mbarara, a malaria test should be done:	
Only when a child has a fever and a stiff neck	
Never, you can presume the fever is due to malaria	
In all cases when the child has a fever with or without a general danger signs	
Only when the child has not taken malaria treatment	
In children without other diagnosis	

Answer with 0 if you strongly disagree, 1 if you are neither agree nor disagree 2 if you strongly agree	
10. What is a general danger sign?	
A sign that a sick young infant is ill	
A sign that the child is beginning to get sick, so the mother should watch him more carefully at home	
A sign of very serious illness in a sick child, which requires urgent referral	
A sign that the child most likely has pneumonia	
A sign that the child has been sick for a long time	

Quality of care for common childhood infections study

Participant number: |____|_____|______|

Highest level of Training: Tick one that applies

Nursing assistant	Certificate in Nurse	Diploma in nurse/midwifery	Degree nurse/midwife	Diploma in Clinical medicine	Doctor (MBChB)	Master degree in paediatrics or other clinical discipline
